# Survival curve identifies critical period for postoperative mortality in cardiac patients undergoing emergency general surgery

**DOI:** 10.1038/s41598-020-72647-7

**Published:** 2020-09-23

**Authors:** Diego Ramos Martines, Fernanda Nii, Kayo Augusto de Almeida Medeiros, Bárbara Justo Carvalho, Leonardo Zumerkorn Pipek, Gustavo Heluani Antunes de Mesquita, Leandro Ryuchi Iuamoto, Gustavo B. F. Oliveira, Antonio Carlos Mugayar Bianco, Alberto Meyer

**Affiliations:** 1 Faculdade de Medicina FMUSP, Universidade de São Paulo, São Paulo, Brazil; 2 Cardiac Care Unit, Dante Pazzanese Institute of Cardiology, São Paulo, Brazil; 3 Departamento de Gastroenterologia, Faculdade de Medicina, Hospital das Clínicas HCFMUSP, Universidade de São Paulo, São Paulo, Brazil; 4 General and Gastrointestinal Surgery, Dante Pazzanese Institute of Cardiology, São Paulo, Brazil

**Keywords:** Cardiology, Gastroenterology

## Abstract

The number of non-cardiac major surgeries carried out has significantly increased in recent years to around 200 million procedures carried out annually. Approximately 30% of patients submitted to non-cardiac surgery present some form of cardiovascular comorbidity. In emergency situations, with less surgery planning time and greater clinical severity, the risks become even more significant. The aim of this study is to determine the incidence and clinical outcomes in patients with cardiovascular disease submitted to non-cardiac surgical procedures in a single cardiovascular referral center. This is a prospective cohort study of patients with cardiovascular disease submitted to non-cardiovascular surgery. All procedures were carried out by the same surgeon, between January 2006 and January 2018. 240 patients included were elderly, 154 were male (64%), 8 patients presented two diagnoses. Of the resulting 248 procedures carried out, 230 were emergency (92.8%). From the data obtained it was possible to estimate the day from which the occurrence of mortality is less probable in the postoperative phase. Our research evaluated the epidemiological profile of the surgeries and we were able to estimate the survival and delimit the period of greatest risk of mortality in these patients. The high rate of acute mesenteric ischemia was notable, a serious and frequently fatal condition.

## Introduction

The number of non-cardiac major surgeries carried out has significantly increased in recent years to around 200 million procedures carried out annually^1^. This type of surgery exposes patients to risks such as hemorrhage, inflammatory systemic responses, infection, pulmonary embolism and acute myocardial infarction, which can lead to acute overload of the cardiovascular system. According to European Society of Cardiology and European Society of Anaesthesiology data, approximately 30% of patients submitted to non-cardiac surgery present some form of cardiovascular comorbidity^2^, with a resulting greater risk of complications or a fatal cardiovascular event in the postoperative period.

In emergency situations, with less surgery planning time and greater clinical severity, the risks become even more significant, multiplying the incidence of myocardial infarction or mortality because of postoperative cardiovascular conditions by between 2.5 and 4 times^3^. This increased risk can be identified by the American Society of Anesthesiologists (ASA)^4^ score, among others.

In 2014, the American College of Cardiology/American Heart Association (ACC/AHA) Task Force for Practice Guidelines published a review of its recommendations for evaluation of cardiovascular risk in patients submitted to non-cardiac procedures^5^. As more surgical procedures take place in elderly patients, anesthesiologists will more often face patients with diagnosed or even suspected chronic heart failure in the perioperative period^6^. Patients with chronic heart failure form a distinct risk population, which is associated with increased risk in the perioperative period. These initiatives show the relevance of the clinical conditions found in cardiopathic patients and their evolution in the postoperative period.

The aim of this study is to determine the incidence and clinical outcomes in patients with cardiovascular disease submitted to non-cardiac surgical procedures in a single cardiovascular referral center.

## Methods

This is a prospective cohort study of patients with cardiovascular disease submitted to non-cardiovascular surgery. All procedures were carried out by the same surgeon, between January 2006 and January 2018, in the General Surgery Service at the Dante Pazzanese Institute of Cardiology (DPIC). The information related to the surgical procedures was taken from a database and complemented with an analysis of medical records.

The variables analyzed were sex, age, clinical diagnosis, surgical indication, surgical procedures carried out, short term mortality (up to 30 days postoperative).

### Inclusion criteria

The patients included in this study were over the age of 18, with a cardiovascular disease referred for surgical non-cardiac treatment at the institution, either in emergency or for elective surgery.

### Exclusion criteria

There were no specific exclusion criteria, given that the aim was to report all adult patients that had a documented diagnosis and indication for a non-cardiovascular surgical procedure.

### Statistical analysis

A descriptive exploration is presented in frequency tables for qualitative variables, including the confidence intervals of the proportions. *P* < 0.05 was regarded as statistically significant.

Univariate Cox analysis was first performed to analyzed whether the variables sex and diseases were significantly associated with overall survival (OS) in cardiac patients undergoing general surgery using the “survival” package. The OS was calculated from the time of diagnosis to death from any cause. A Kaplan–Meier survival curve was constructed to describe the survival of patients with treatments for different diseases. For inter-group comparison regarding sex and treatments, the Log-Rank (Mantel-Cox) test was used. All significance tests were two-tailed and with a confidence interval of 95%.

The analyses were performed using the statistical software RStudio Team (2015). RStudio: Integrated Development for R. RStudio, Inc., Boston, MA.

### Ethical considerations

This study was approved by the Ethics Committee of the Dante Pazzanese Institute of Cardiology and all patients consented to participate and signed the Free and Informed Consent Form. All procedures performed in this study were in accordance with the ethical standards of the institutional research committee and with the 1964 Helsinki Declaration and its later amendments or comparable ethical standards.

### Consent for publication

All authors consented for publication.

## Results

All the 240 patients included were elderly, 154 were male (64%), 8 patients presented two diagnoses. Of the resulting 248 procedures carried out, 230 were emergency (92.8%). The most common diagnoses were: acute mesenteric ischemia (53), acute cholecystitis (46), acute appendicitis (25), Fournier syndrome (24), emergency hernia (19), elective hernia (18), perforated peptic ulcer (9), colonic neoplasia (8). Other diagnoses comprised 46 procedures ( 18.5%) (Table 1).

**Table 1:** Other diagnosis (number of procedures).

Infected hematoma (5)	Gastric neoplasia (4)	Choledocholithiasis (3)
Active obstructive abdomen (3)	Enteric fistula (2)	Ovarian neoplasia (1)
Ovarian cyst (1)	Intestinal perforation (1)	Esophageal obstruction (1)
Hepatic neoplasia (1)	Biliary neoplasia (1)	Liver failure (1)
Splenic trauma (1)	Lymphoma (1)	Hemo retroperitonitis (1)
Splenic aneurysm (1)	Meckel’s diverticulum (1)	

We considered short term mortality as less than 30 days after surgical intervention. As an overall result, postoperative mortality was 30% (72 cases). The diagnoses with the highest mortalities were acute mesenteric ischemia (71.7%), perforated peptic ulcer (66.6%) and colonic neoplasia (37.5%). Other diagnoses also present significant mortality (Table 2), except for elective hernias (no mortalities).

**Table 2:** Distribution of mortality by diagnosis.

	Live		Death within 30 days		Death after 30 days	
	N	%	N	%	N	%
Acute mesenteric ischemia	14	26.4	38	71.7	1	1.9
Acute cholecystitis	40	87	3	6.5	3	6.5
Acute appendicitis	20	80	3	12	2	8
Fournier	15	62.5	4	16.7	5	20.8
Emergency hernia	11	57.9	6	31.6	2	10.5
Perforated peptic ulcer	3	33.3	6	66.7	0	0.0
Colon neoplasia	5	62.5	3	37.5	0	0.0
Other diagnoses	49	76.6	9	14	6	9.3

**Figure 1:**
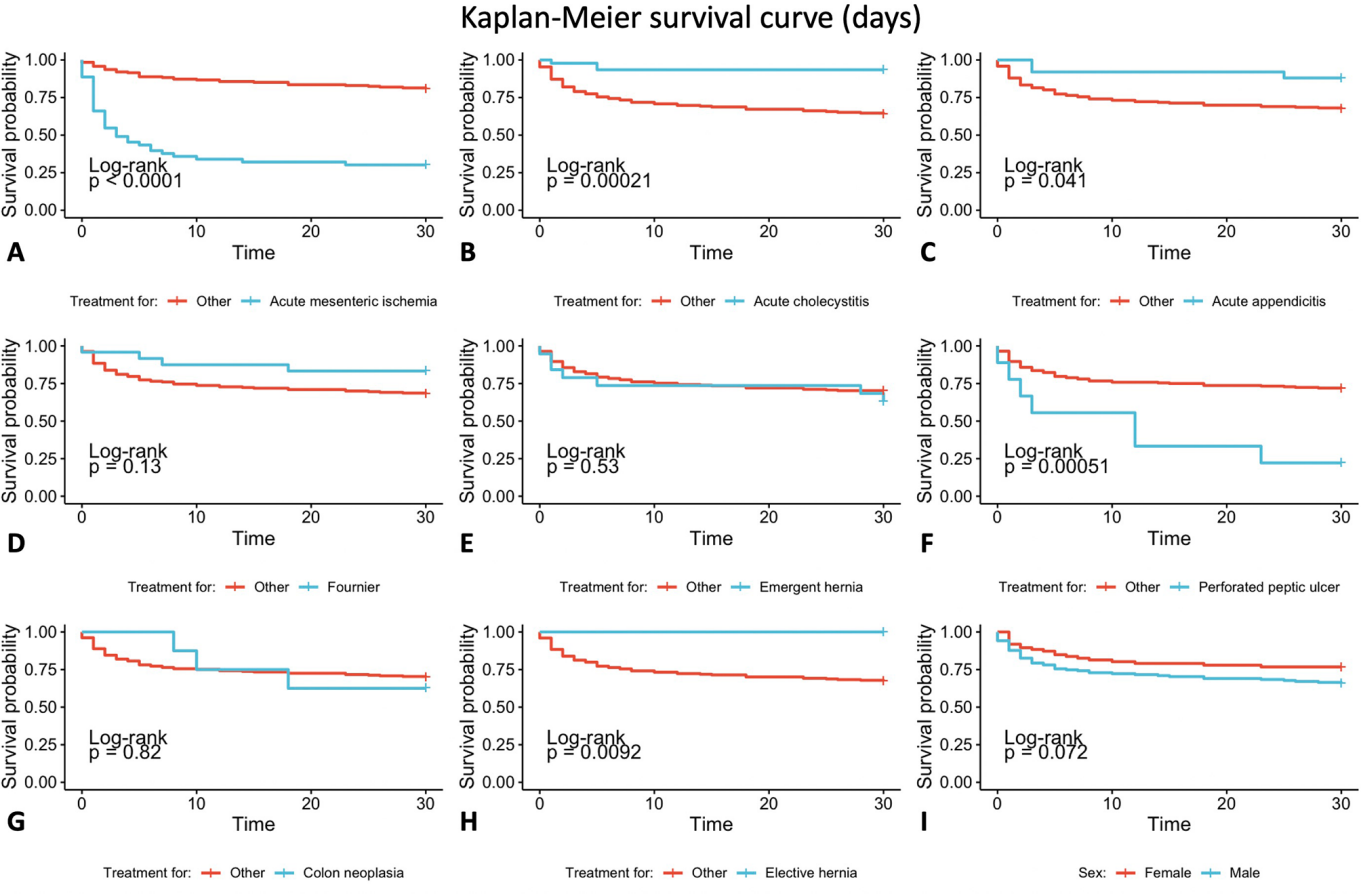
30-day survival curve in patients with the most common diagnoses and comparison with treatment for other diagnoses. (**A**) Acute mesenteric ischemia. (**B**) Acute cholecystitis. (**C**) Acute appendicitis. (**D**) Fournier. (**E**) Emergent hernia. (**F**) Perforated peptic ulcer. (**G**) Colon neoplasia. (**H**) Elective hernia. (**I**) Overall mortality comparison between sex.

The Kaplan–Meier mortality curve for each procedure was analyzed and a Log-Rank comparison showed no significant differences in postoperative mortality between the sexes for any procedure (p > 0.05). There was also no significant difference in the overall mortality comparing male and female (p > 0.05) Fig. 1.

**Figure 2:**
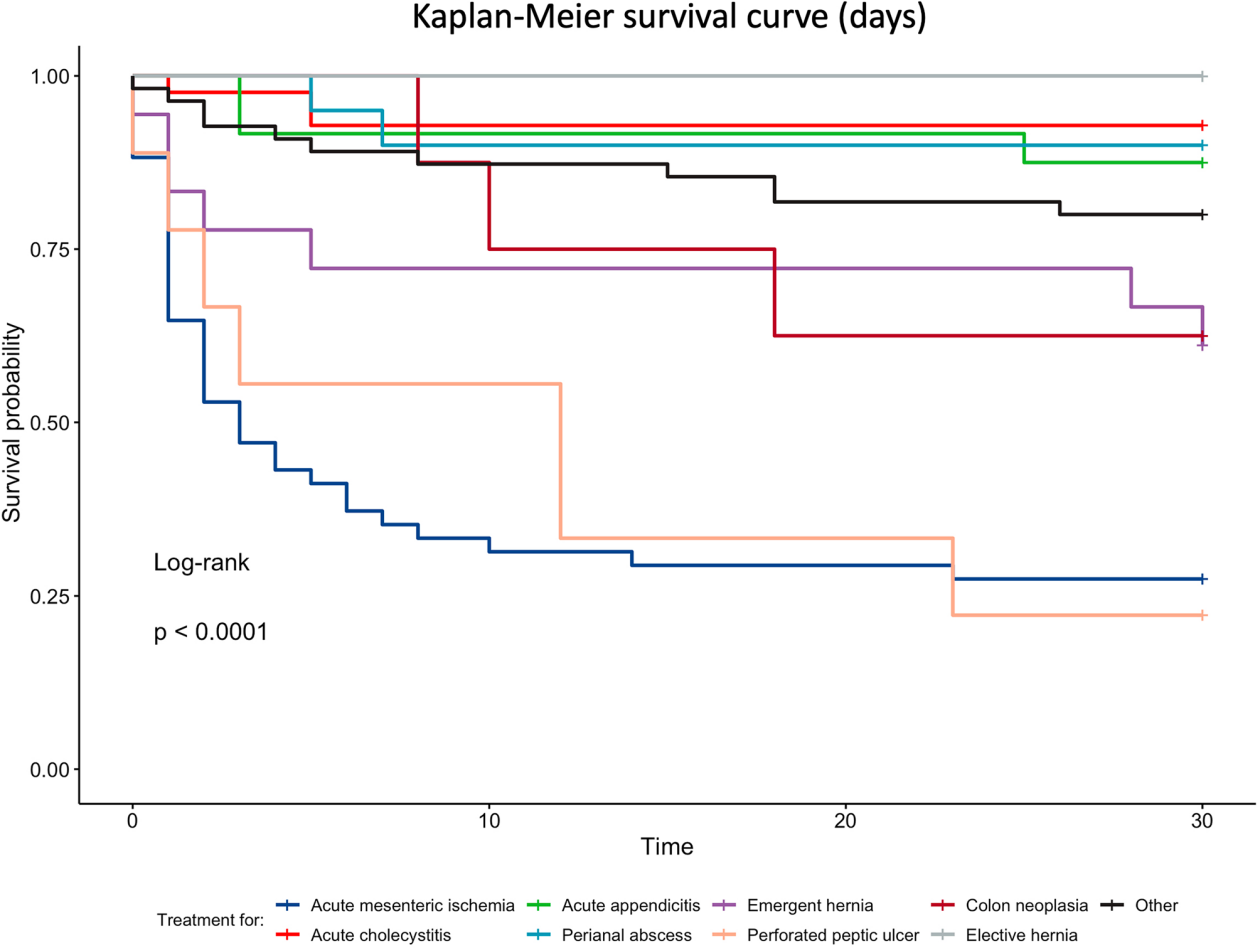
30 day survival curve in patients with the most common diagnoses.

The mortality for the treatment of the eight most common diagnosis was individually analyzed with the Kaplan–Meier method and compared to the overall mortality with Log-Rank test—Fig. 1. Figure 2 shows a Kaplan–Meier curve comparing the mortality for the eight most common diagnosis.

From the data obtained it was possible to estimate the day from which the occurrence of mortality is less probable in the postoperative phase (Table 3) and the necessary days to stabilize the survival curve (Fig. 1). The statistical analyses also shows that some treatments have a significant higher mortality when compared to the overall mortality for cardiac patients undergoing general surgery, including acute mesenteric ischemia and perforated peptic ulcer. On the other hand, treatment for acute cholecystitis and elective hernia have a lower mortality when compared to other treatments.

**Table 3:** Estimate in days of fatal events during the postoperative period and Log-Rank comparison with overall mortality.

Diagnosis	Estimate	Standard error	95% confidence interval		Log-Rank
			Lower limit	Upper limit	p-value
Acute mesenteric ischemia	11.3	1.764	7.9	14.8	< 0.0001
Acute cholecystitis	28.3	0.961	26.4	30.2	0.00021
Acute appendicitis	27.6	1.466	24.8	30.5	0.041
Fournier	26.3	1.797	22.7	29.8	0.13
Emergency hernia	23.8	2.919	18.1	29.5	0.53
Perforated peptic ulcer	12.6	3.869	5	20.1	0.00051
Colon neoplasia	23.3	3.22	16.9	29.6	0.82

## Discussion

In our study, an analysis of surgical procedures was carried out and the mortality was estimated in procedures of varying complexity, from exploratory laparotomy to elective hernioplasty. Many of the interventions (92.8%) were emergency surgery, which represents a risk factor for 30-day mortality.

The emergency procedures present significant numbers. In the United States, for example, there is an annual mean of around 3 million emergency surgeries carried out^7^. In this context, Reinke et al., carried out a study assessing hospitalization for non-cardiac general surgery^8^. The most common surgical procedures were cholecystectomy, wound debridement, partial resection of the small intestine and appendectomy, while in our study the most prevalent were exploratory laparotomy, cholecystectomy, appendectomy and wound debridement of perineal abscesses. A correspondence was verified between the cases in our study and those reported in others with similar risk factors and comorbidities^8,9^.

Despite the higher number of patients with high cardiovascular risk, the mortality obtained in the American study in patients submitted to emergency surgery was 3.8%^9^, a much lower number than that found in our study. This contrast could be due to the severity of the conditions the patients presented in our center and the elevated rate of acute mesenteric ischemia (AMI). With 53 occurrences, this diagnosis was responsible for 52.7% of the mortality in our study.

AMI is caused by a sudden interruption of the blood supply for a segment of the small intestine, causing ischemia, cellular damage and intestinal necrosis. Most cases are caused by embolic occlusion (40 to 50%) or by thrombotic occlusion (25 to 35%). Mesenteric embolisms are associated with cardiac complications such as atrial fibrillation, left ventricle myocardial dysfunction and reduced ejection fraction or cardiac valves damaged by endocarditis. AMI is an uncommon cause of abdominal pain, corresponding to less than 0.1% of hospitalizations in a general hospital, but its mortality is between 60 to 80%^10^.

In a recently published article, Gilshtein et al. identified 63 patients with ischemic colitis, the most common form of AMI, in one tertiary center^11^. Most patients were treated clinically, and only the 20% most serious cases, with complications such as peritonitis, hemodynamic instability and untreatable disease, received surgical intervention. These patients had a mortality of 50%, lower than our study (71.7%), which reinforces the gravity of the condition of the patients referred to the center in our study.

From our statistical analysis it was possible to estimate that for AMI, mortality occurs until a mean of 11 days, which represents more than 90% of the deaths from this condition. This information could affect the management of the patient, as it defines the phase of greatest risk.

Another diagnosis that was relevant to our study was the perforated peptic ulcer. Complications of the ulcer, as well as perforation, include bleeding and obstruction. When perforated, the ulcer must be referred to emergency surgical treatment, and is associated with a 30% mortality rate in 30 days^12,13^. Among our patients, a significantly higher mortality rate of 66.7% was observed, probably due to the severity of the condition of patients referred to the center. Furthermore, it was possible to identify that patients tended to die up to the 12th postoperative day. The large CI of 95% (between 5 and 20 days) was a result of our small sample with just 9 patients.

Acute cholecystitis and acute appendicitis were also highly prevalent in our study, but the 30-day mortality of both was low, which resulted in a survival curve with little predictive value.

Similarly, the other diagnoses did not collect enough cases, which shows a need for other studies with a larger sample of patients to obtain significant results. This would allow the period of greatest risk to patients to be identified, and allow care to be tailored accordingly, as with AMI.

## Conclusion

Our research evaluated the epidemiological profile of the surgeries carried out in DPIC and we were able to estimate the survival and delimit the period of greatest risk of mortality in these patients. The high rate of AMI was notable, a serious and frequently fatal condition.

## Abbreviations

ASA: American Society of Anesthesiologists
ACC/AHA: American College of Cardiology/American Heart Association
DPIC: Dante Pazzanese Institute of Cardiology
AMI: Acute mesenteric ischemia

## References

[1] Devereaux PJ, Sessler DI (2015). Cardiac complications in patients undergoing major noncardiac surgery. N. Engl. J. Med..

[2] Kristensen SD, Knuuti J, Saraste A, Anker S, Bøtker HE, De Hert S, Ford I, Gonzalez Juanatey JR, Gorenek B, Heyndrickx GR, Hoeft A, Huber K, Iung B, Kjeldsen KP, Longrois D, Luescher TF, Pierard L, Pocock S, Price S, Roffi M, Sirnes PA, Uva MS, Voudris V, Funck-Brentano C, Authors/Task Force Members (2014). 2014 ESC/ESA Guidelines on non-cardiac surgery: Cardiovascular assessment and management: The Joint Task Force on non-cardiac surgery: Cardiovascular assessment and management of the European Society of Cardiology (ESC) and the European Society of Anaesthesiology (ESA). Eur J Anaesthesiol..

[3] Smilowitz NR, Gupta N, Guo Y, Berger JS, Bangalore S (2017). Perioperative acute myocardial infarction associated with non-cardiac surgery. Eur. Heart J..

[4] de Nadal M, Pérez-Hoyos S, Montejo-González JC, Pearse R, Aldecoa C (2018). European surgical outcomes study (EuSOS) in Spain Intensive care admission and hospital mortality in the elderly after non-cardiac surgery. Med. Intens..

[5] Kaw R, Nagarajan V, Jaikumar L, Halkar M, Mohananey D, Hernandez AV, Ramakrishna H, Wijeysundera D (2019). Predictive value of stress testing, revised cardiac risk index, and functional status in patients undergoing noncardiac surgery. J. Cardiothorac. Vasc. Anesth..

[6] Smit-Fun V, Buhre WF (2016). The patient with chronic heart failure undergoing surgery. Curr. Opin. Anaesthesiol..

[7] Gale SC, Shafi S, Dombrovskiy VY, Arumugam D, Crystal JS (2014). The public health burden of emergency general surgery in the United States: A 10-year analysis of the nationwide inpatient sample—2001 to 2010. J. Trauma Acute Care Surg..

[8] Reinke CE, Thomason M, Paton L, Schiffern L, Rozario N, Matthews BD (2017). Emergency general surgery transfers in the United States: A 10-year analysis. J. Surg. Res..

[9] Ingraham A, Wang X, Havlena J, Hanlon B, Saucke M, Schumacher J, Fernandes-Taylor S, Greenberg C (2019). Factors associated with the interhospital transfer of emergency general surgery patients. J. Surg. Res..

[10] Clair DG, Beach JM (2016). Mesenteric ischemia. N. Engl. J. Med..

[11] Gilshtein H, Hallon K, Kluger Y (2018). Ischemic colitis caused increased early and delayed mortality. World J. Emerg. Surg..

[12] Søreide K, Thorsen K, Harrison EM, Bingener J, Møller MH, Ohene-Yeboah M, Søreide JA (2015). Perforated peptic ulcer. Lancet.

[13] Møller MH, Engebjerg MC, Adamsen S, Bendix J, Thomsen RW (2012). The peptic ulcer perforation (PULP) score: A predictor of mortality following peptic ulcer perforation. A cohort study. Acta Anaesthesiol. Scand..

